# Lithography-free fabrication of silicon nanowire and nanohole arrays by metal-assisted chemical etching

**DOI:** 10.1186/1556-276X-8-155

**Published:** 2013-04-04

**Authors:** Ruiyuan Liu, Fute Zhang, Celal Con, Bo Cui, Baoquan Sun

**Affiliations:** 1 Jiangsu Key Laboratory for Carbon-Based Functional Materials & Devices, Institute of Functional Nano & Soft Materials (FUNSOM), Soochow University, Suzhou, 215123, People's Republic of China; 2 Department of Electrical and Computer Engineering, University of Waterloo, 200 University Avenue, West Waterloo, Ontario, N2L3G1, Canada

**Keywords:** Metal-assisted chemical etching, Silicon nanowire arrays, Silicon nanohole arrays, Silver thin film dewetting

## Abstract

We demonstrated a novel, simple, and low-cost method to fabricate silicon nanowire (SiNW) arrays and silicon nanohole (SiNH) arrays based on thin silver (Ag) film dewetting process combined with metal-assisted chemical etching. Ag mesh with holes and semispherical Ag nanoparticles can be prepared by simple thermal annealing of Ag thin film on a silicon substrate. Both the diameter and the distribution of mesh holes as well as the nanoparticles can be manipulated by the film thickness and the annealing temperature. The silicon underneath Ag coverage was etched off with the catalysis of metal in an aqueous solution containing HF and an oxidant, which form silicon nanostructures (either SiNW or SiNH arrays). The morphologies of the corresponding etched SiNW and SiNH arrays matched well with that of Ag holes and nanoparticles. This novel method allows lithography-free fabrication of the SiNW and SiNH arrays with control of the size and distribution.

## Background

Silicon nanostructures such as silicon nanowire (SiNW), nanocone, or nanohole (SiNH) arrays have attracted intensive attention due to their unique optical, electrical, and thermal properties for promising applications in the fields of solar cells [1-6], field-effect transistors [7], as well as chemical and biological sensors [8,9]. Besides their intrinsic characteristics inherited from bulk silicon, the morphologies and distribution of the nanostructures play a dominant role on their properties. As for both the basic studies and applications of SiNW arrays, precise control of the diameter, the length, the density, and the surface are of vital importance. To achieve large-area vertically aligned SiNW arrays with high uniformity, it is very popular to apply metal-assisted chemical etching (MaCE) as a low-cost etching method [6,10-12]. In this method, a thin noble metal film with arrays of holes is formed on a silicon substrate and then the silicon underneath the metal is etched off with the catalysis of metal in an aqueous solution containing HF and an oxidant, leaving behind arrays of SiNW whose distribution and diameter are determined by the metal film. To prepare a metal film with good ordered arrays of nanoholes, nanosphere lithography [2,13,14], interference lithography [15,16], block copolymers [17], or anodic aluminum oxide [18-20] has been extensively adopted. Though SiNW arrays with well-controlled diameter, length, and density have been achieved, complicated processing steps are involved prior to MaCE. The fabrication of SiNH array structure also faces the same issues. In addition, specific techniques such as deep ultraviolet lithography are also required in order to achieve high-quality periodic SiNH arrays [4,21]. In this work, we present a facile method to fabricate SiNW arrays as well as SiNH arrays based on metal film dewetting process, which dramatically simplifies the fabrication process by avoiding complicated lithography patterning process. The patterned silver (Ag) structure can be tuned by varying the thickness of the Ag film and annealing temperature on the silicon substrate. With the control of the annealing process, metal film with arrays of holes or nanoparticles can be generated on the substrate. The silicon underneath the silver is etched off, thus SiNW or SiNH arrays can be achieved by MaCE with the catalysis of the metal. The as-fabricated Si nanostructures match well with the self-patterned metal structure.

## Methods

**Figure 1:**
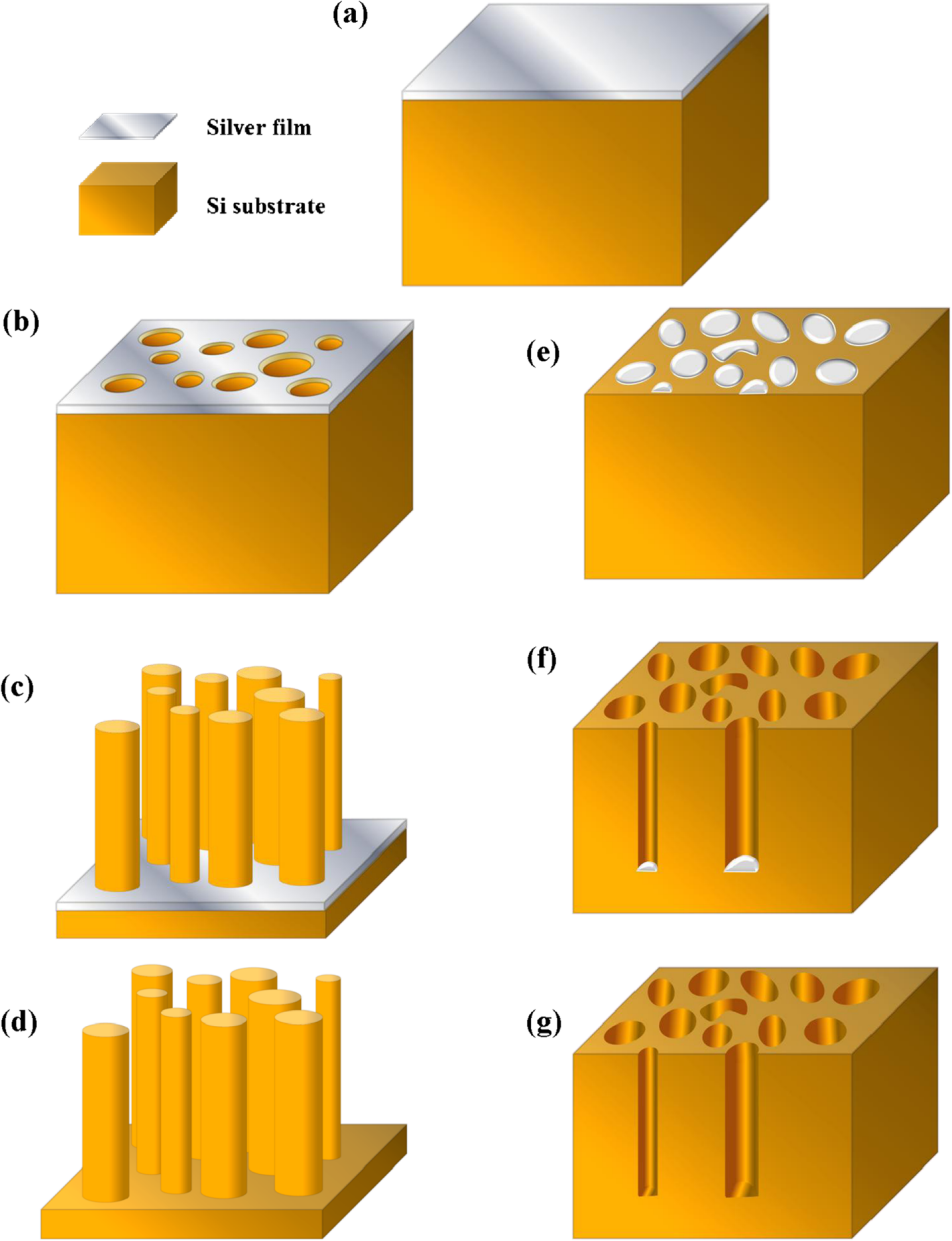
**Schematic of the SiNW and SiNH array fabrication process.** (**a**) Ag film is fabricated by thermal evaporation on a Si substrate. (**b**) Ag film with regular holes after relatively low-temperature thermal treatment. (**c**, **d**) SiNW arrays achieved after MaCE corresponding to (**b**). (**e**) Ag nanoparticles with uniform shape after relatively high-temperature thermal treatment. (**f**, **g**) SiNH arrays achieved after MaCE corresponding to (**d**).

The fabrication process of the SiNW and the SiNH arrays is illustrated in Figure 1. Typically, n-type (100) silicon wafers (resistivity, 7 ~ 9 Ω cm) were used as the substrate. Silicon wafers were cleaned in acetone, ethanol, and deionized water for 20 min subsequently. Then, the wafers were cleaned in a boiling piranha solution (3:1 (*v*/*v*) H_2_SO_4_/H_2_O_2_, 110°C, 1 h) to remove any organic residue. After thoroughly rinsed with deionized water, the silicon substrate was immersed into 5 M HF solution for 10 min, leading to H-terminated silicon surface. Then, the substrates were rinsed for several times with deionized water and dried under N_2_ airflow. Ag films with different thicknesses (8 ~ 30 nm) were deposited onto the cleaned H-Si substrate by thermal evaporation (Figure 1a). For a thin Ag film, with increasing annealing temperatures, the morphologies of the Ag film transform from continuous flat film to mesh one with nanoholes (Figure 1b), bi-continuous structures, and finally nanoparticles (Figure 1d). Then, SiNW and SiNH arrays could be achieved by immersing the Ag-covered Si substrate into a mixed etchant solution consisting of HF and H_2_O_2_, with the catalysis of either the Ag mesh or the Ag nanoparticles, respectively (Figure 1c,f).

## Results and discussion

### Dewetting process of Ag films

Dewetting process of thin film on a solid substrate has been well investigated in the past decades [22-25]. Solid films are usually metastable or unstable in the as-deposited state, and they will spontaneously dewet or agglomerate to form islands when heated to certain temperatures at which the mobility of the constituent atoms is sufficiently high. Dewetting occurs at the holes preexisting during the deposition process (as in this case), at film edges, or at newly formed holes, which is overall a hole nucleation and growth phenomena. Whatever their source is, a process that leads to hole formation in a film is a prerequisite for dewetting where the holes could potentially serve as nucleation sites or as nuclei themselves [23]. The most common origin for the heterogeneous nucleation is grain boundary grooving which may occur from the free surface of the film and the film/substrate interface. Hole formation would be most likely when the grain boundary grooves grow sufficiently large. The formation and growth of these holes takes an incubation time for dewetting that depends on film thickness. Hole formation can also occur by grain sinking that results from a diffusional flow when a lower tensile grain loses material to a higher tensile one [23]. Whether the initial holes are developed by grain grooving, grain sinking, or just deposition process, the overall dewetting process is determined by the growth of the holes. As the holes grow, the development of rims slows down the rate of edge retraction by reducing the strain energy of the system. At the early stage, small circular holes grow immediately until neighboring holes meet and form common rims of networks, and new holes may still continue to form throughout the dewetting process. The networks finally become unstable and break up into stable islands with minimum local energy state via the Rayleigh instability.

**Figure 2:**
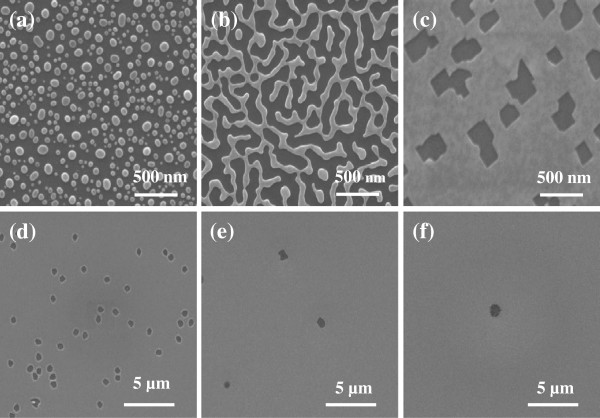
**SEM images of morphologies of different Ag film thicknesses annealed at 150°C for 10 min.** (**a**) 9, (**b**), 11, (**c**) 14, (**d**) 16, (**e**) 20, and (**f**) 29 nm.

**Figure 3:**
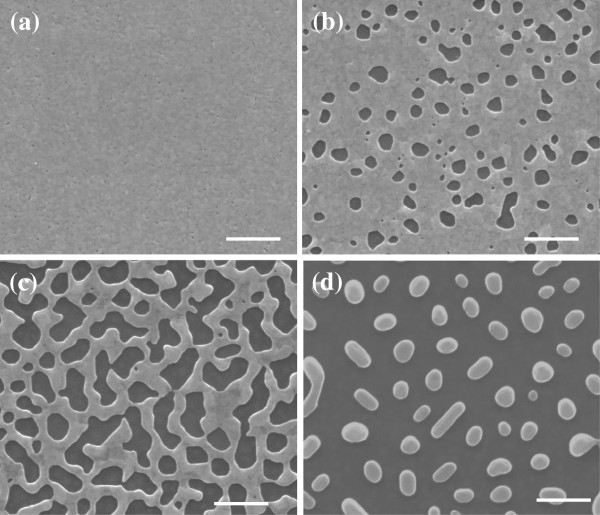
**The morphology of 16-nm silver film annealed at different temperatures for 10 min.** (**a**) Unannealed, (**b**) 150°C, (**c**) 200°C, and (**d**) 250°C. All scale bars are 500 nm.

Here, the Ag layer dewetting morphology was investigated on Si substrate as a function of film thickness, which ranged from 7 to 41 nm. Different annealing temperatures from to 300°C were utilized to explore the dewetting behavior. In order to investigate the influence of the Ag film thickness on the morphologies during the thermal dewetting process, Ag films of 9, 11, 14, 16, 20, and 29 nm were annealed at 150°C for 10 min in inert atmosphere (Figure 2). As shown in Figure 2, for a given energy (at a fixed annealing temperature), the morphology is apparently different for different film thicknesses. In Figure 2a, the 9-nm-thick Ag film has completely converted from flat film to nanoparticle state, and bi-continuous structures can be observed in the 11-nm-thick one (Figure 2b). On the contrary, hardly any hole can be observed when the thickness is above 20 nm (Figure 2f), which can be attributed to the film thickness-dependent intermolecular forces. It was also confirmed in our experiment that only Ag films in the range of 10 to 20 nm could generate well-distributed Ag network structure at a moderate temperature (approximately 150°C) [25]. Otherwise, a higher annealing temperature is indispensable to achieve Ag mesh (Figure 3). It means that the temperature at which dewetting occurs increases with increasing metal film thickness. This is critical for our later step either to form SiNW arrays utilizing the Ag mesh film with holes or to form SiNH arrays utilizing Ag nanoparticles. In other words, the energy required to get a morphology transition for various film thicknesses is different, and with increasing thicknesses of the film, the required temperature/energy to form the metal mesh increased.

Meantime, for a given film thickness (e.g., 16 nm), as the annealing temperature increases gradually, the morphologies of the film transfer from compact film to mesh one with circular or quadrate holes (Figure 3b) and finally to isolated Ag semispherical nanoparticles (Figure 3d). If the film is thin enough (e.g., 5 nm), only isolated island can be achieved even at a very low annealing temperature, which may originate from the initial uncontinuous feature during the deposition process. If the film is too thick (e.g., 41 nm), no obvious hole can be observed even for annealing temperature as high as 300°C. The dependence of morphologies on the film thickness displays a similar behavior. To a certain degree, the same morphology can be achieved with different combinations of film thickness and annealing temperature.

### Fabrication of SiNW arrays utilizing Ag meshes

**Figure 4:**
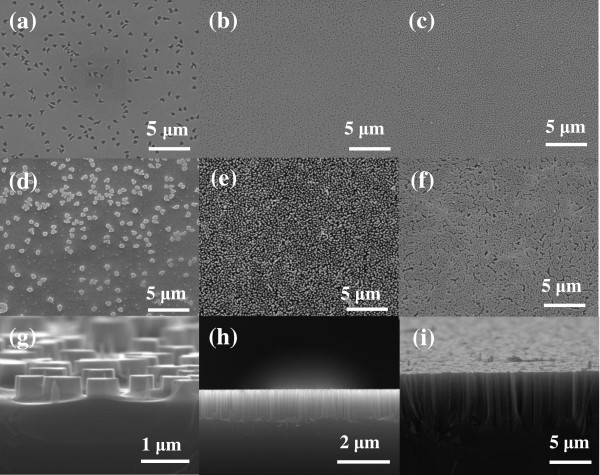
**SEM images of films with different thicknesses and annealing temperatures and corresponding etching results.** (**a**) The 11-nm-thick Ag film on Si substrate annealed at 120°C for 10 min. (**b**) The 12-nm-thick Ag film on Si substrate annealed at 160°C for 10 min. (**c**) The 13-nm-thick Ag film on Si substrate annealed at 175°C for 10 min. Planar and cross-sectional images of their corresponding etched substrate: (**d**, **g**) corresponding to (**a**), (**e**, **h**) corresponding to (**b**), and (**f**, **i**) corresponding to (**c**).

Figure 4a,b,c shows the morphologies of dewetting Ag films with different hole sizes. By tuning the film thickness and annealing temperature, the density and the diameters of the holes can be readily controlled. With Ag mesh patterned as catalyst on silicon substrate, fabrication of vertical (100) SiNW arrays with controlled morphologies were achieved, as shown in Figure 4. It is evident that the morphology of SiNWs matches well with the shape of the corresponding holes on the Ag films. It is interesting that not only circular (Figure 4b,c) but also quadrate (Figure 4a) cross-sectional SiNWs can be formed using this method. The slight mismatch between the Ag films and the corresponding SiNWs can be attributed to the gradual erosion of the ultrathin Ag film during the etching [18].

**Figure 5:**
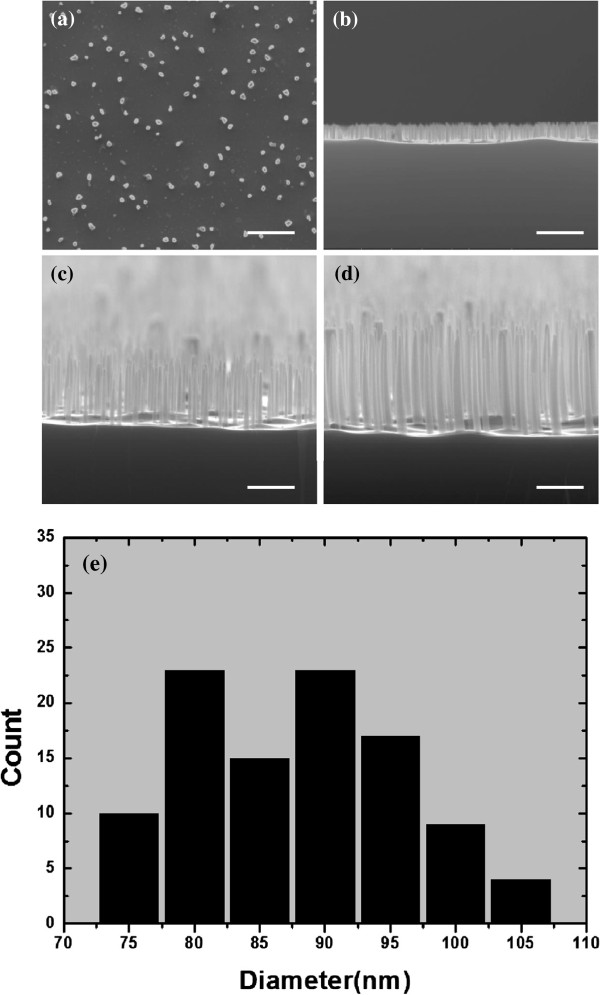
**SEM images of plane-view SiNW arrays, cross-sectional SEM images of the SiNWs, and statistical distribution.** (**a**) SEM images of plane-view SiNW arrays achieved with the catalysis of a 14-nm-thick Ag film annealed at 150°C for 10 min and cross-sectional SEM images of the SiNWs etched for (**b**) 5 min (nanowire length 0.5 μm), (**c**) 10 min (1 μm), and (**d**) 20 min (2 μm). All scale bars are 500 nm. (**e**) The statistical distribution for the average diameters of the corresponding SiNWs.

Another important parameter of the SiNW arrays is the length, which can be controlled by varying the etching time. Figure 5b,c,d shows the cross-sectional scanning electron microscope (SEM) images of SiNW arrays fabricated with etching times of 5, 10, and 20 min, respectively. The Ag film is 14 nm and annealed at 150°C for 10 min. As a result, nanowires with lengths of about 0.5 μm, about 1 μm, and about 2 μm are achieved, respectively. The length of the nanowires shows good linear relationship with the duration of the etching time. The statistical analysis (Figure 5e) shows the good diameter distribution of the as-fabricated SiNWs. Here, the tapered morphology of the nanowires resulted from the gradual Ag dissolution-induced hole size increase.

### Fabrication of SiNH arrays utilizing Ag nanoparticles

**Figure 6:**
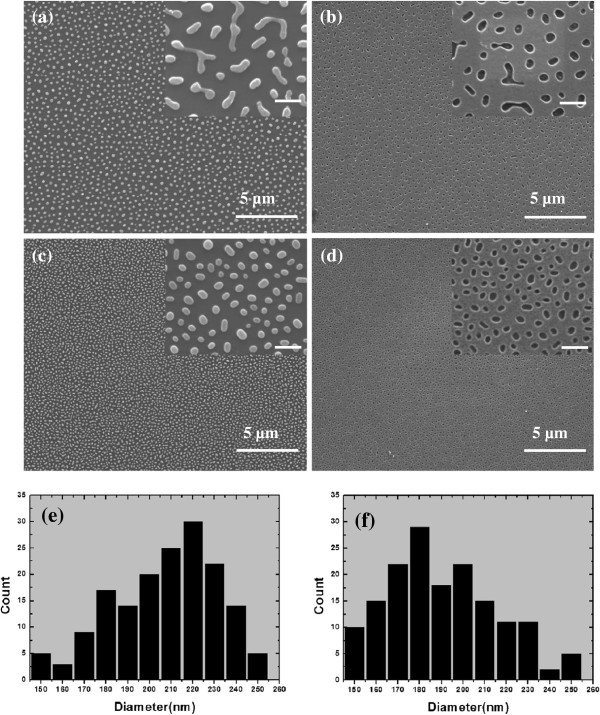
**SEM images of Ag film.** (**a**) A 12-nm Ag film annealed at 200°C for 10 min, (**b**) planar view of corresponding etching results to (**a**), (**c**) 14-nm-thick Ag film annealed at 250°C for 10 min, and (**d**) planar view of corresponding etching results to (**c**). All the scale bars of the insets are 500 nm. (**e, f**) The statistical distribution for the average hole diameters for (**b**) and (**d**), respectively.

When the metal film is annealed at higher temperature, the continuous thin Ag film finally transforms into isolated nanoparticles (Figure 6). As shown in Figure 6a,c, the Ag particles are semispherical and exhibit good distribution and uniformity. The parameters of the nanoparticles can be tuned by varying the film thickness and annealing temperature. When the film is as thin as 9 nm or below, only small dense nanoparticles can be achieved regardless of the annealing temperatures (Figure 2a). At this stage, the morphology of the annealed film seems to be dominated by the initial morphology of deposited metal film. For the thickness between 10 and 20 nm (e.g., 12 and 14 nm), the annealing temperature obviously influences the shape, diameter, and center-to-center distance of the nanoparticles (Figure 6a,c). The variation in density of the nanoparticles (Figure 6e,f) is attributed to the different Ag quantities or thicknesses. Relevant work has been previously reported by Wang et al. [26] who manipulated the size and distribution of Ag nanoparticles by the film thickness and laser ablation parameters. However, they only studied the influence of film thickness without a more detailed experiment. Here, our investigation shows that the nanoparticles are irregular before the thorough breaking up of the bi-continuous structure. Then, they tend to be more and more spherical with the increasing annealing temperature, and finally, most strip-type nanoparticles are transformed into perfectly spherical shapes due to the high surface energy of metal. Once stable semispherical nanoparticles are formed, the morphology rarely changes even at high annealing temperatures from 200°C to 300°C. With the semispherical Ag nanoparticles patterned on the Si substrate as catalyst, SiNH arrays can be fabricated by chemical etching. As is shown in Figure 6b,d, the morphologies of SiNH arrays match well with the corresponding Ag nanoparticles shown in Figure 6a,c, respectively. It has been pointed out that the light-trapping characteristics of the SiNH arrays were comparable to or even better than nanorods [27]. A maximum efficiency of 27.8% from Si nanohole solar cells was predicted by optimizing various structural parameters.

## Conclusion

We demonstrate a simple and low-cost method based on the metal dewetting process combined with Ag-assisted chemical etching to fabricate SiNW and SiNH arrays. Both Ag mesh with holes and Ag nanoparticles can be formed without a lithography step. The morphologies are controlled by the Ag film dewetting behavior via thermal annealing. By adjusting the film thickness and annealing temperature, the size and distribution of the holes and nanoparticles can be manipulated. The morphologies of the as-fabricated SiNW and SiNH arrays match well with the holes and nanoparticles. The density, diameter, and distance between adjacent nanowire (nanohole) can be facilely tuned by controlling the respective Ag mesh (nanoparticle). In addition, the length (depth) of nanowire can be adjusted by the etching time. As a result, this is a simple, mask-free, and cost-effective method to fabricate wafer-sized silicon nanostructures.

## Competing interests

The authors declare that they have no competing interests.

## Authors' contributions

RL designed the experiments and carried out the characterization. FZ participated in the SiNW fabrication. CC and BC made substantial contributions to the conception and design of this paper. RL and BS wrote the paper. All authors read and approved the final manuscript.
